# Letter from Iowa

**Published:** 1881-04

**Authors:** 


### Domestic Correspondence.

---

### ARTICLE IX.

### LETTER FROM IOWA.

EDITORS JOURNAL AND EXAMINER:—On Sunday, Oct. 17, 1880, a party of Russian immigrants, numbering about sixty, arrived in southeastern Dakota. When they reached Sioux City, Iowa, where there was a delay of two or three hours, one of the immigrants was very sick, some said with "ship fever;" others would have it small-pox. While waiting for a train, the unfortunate sick man was the center of an admiring crowd of the curious and inquisitive, who were freely allowed to approach and inspect him. The enlightened health officer of Sioux City, a town of 8,000 souls, did not isolate the man—did not detain him and give him Christian care, but sent him off as fast as possible, although the weather was bitingly cold. In this indecent haste to get rid of a seeming plague, the municipal authority concurred. The poor sick man proceeded in a crowded car, on his journey, of sixty-five miles, and reached Yankton, the capital of Dakota Territory, near which is a Russian settlement, only to die a few days after, but not until he had infected others; but as the cases were concealed and stoutly denied, I cannot give the number.

Either there was gross and culpable ignorance of the nature of the man's malady (he had variola in the vesicular stage), or cruel, criminal, shameful fear. Directly after the departure of the Russians from Sioux City, it was bruited the sick man really had small-pox. A public recommendation by the municipal authorities to get vaccinated, turned suspicion into reality, and then commenced a wild, unreasoning alarm, and a rush for vaccination. Every one wanted to be vaccinated immediately, while there was not enough vaccine virus on hand to vaccinate one-twentieth of the applicants. A brisk traffic in vaccine lymph suddenly started into existence, and people bought lymph, but scab by preference, anywhere and everywhere it could be found, and vaccinated themselves. A certain doctor (?) paid a house-to-house visitation, probably at the suggestion of the health officer, and vaccinated, for twenty-five cents each, all found willing to accept his proffered services. Out of the number vaccinated by this person and of those who vaccinated themselves, there was an unusually large percentage of cases of erysipelas and probably other forms of blood poisoning; and many an arm was rendered useless if not an encumbrance for months. People have learned by this time that they themselves and so-called doctors are not the best to practice vaccination; and many must long remember the small-pox scare in Sioux City, Iowa, during the winter of 1880–81.

A man who acted as interpreter to the Russians during their short stay in Sioux City, in due time manifested small-pox in the confluent form—he was entirely unvaccinated and perhaps ignorant of the nature of the disease from which the Russian suffered—and died within a few days. From this man others caught the infection; in particular one person, not susceptible, who acted as a carrier of the infection from Sioux City to Jefferson, D. T., a distance of fourteen miles, in very cold weather in the month of November, where the disease became epidemic chiefly among Canadian French and half-breeds. Every town in the vicinity of Jefferson, D. T., in Nebraska and in Iowa, set up a supposed rigid quarantine (the writer conversed with some of the quarantined in Sioux City), with a threat of $25 fine for any infringement of the same. No assistance, that I am aware of, was sent to the afflicted people. Two weeks ago the aforesaid twenty five cent doctor (?) was dispatched to the sorely tried community to inquire into the state of affairs, when it was supposed the disease, for want of further pabulum, had expended itself. A "*public meeting*"(*?*) was called at which no one from the infected families or houses was permitted to be present. It was concluded and publicly reported that the quarantine imposed no hardship, no want, no suffering, and this, remember, without affording the quarantined, who alone can tell, an opportunity to say one word! It was recommended to raise the quarantine, because the last reported case of small-pox ended in death eleven days before, and three days before another case had had time to *positively declare itself.* This disciple of Æsculapius carried with him, and left behind for distribution among the afflicted, chloride of lime and muriatic acid, as disinfectants. But he did not enter a single infected house, only leaving these powerful chemicals to clear it of all further deadly germs!

In plain, sober, honest fact, during the prevalence of this epidemic the people among whom the disease prevailed were, like Robinson Crusoe, practically "out of humanity's reach," and it is not yet possible to say what hardships and sufferings they endured. Some bodies were left unburied for many days for want of persons to perform this last sad service, which should have been done immediately.

The returns show that 27 families were attacked, giving a total of 96 cases, with 22 deaths in the township of Jefferson, D. T., alone. There was terrible bungling from beginning to end of this epidemic. No effort was made to *stamp out* the disease which was left to *wear itself out.* A shameful, disgraceful dread was displayed everywhere by those who should do otherwise.

One more disgraceful matter remains to be noticed, and it is this: Why did railroad companies afford means of transportation to any one sick with such a highly infectious disease? And as far as the writer could discover, no disinfection whatever was practiced by the railroad officers—nothing done to protect persons from catching small-pox from *their cars* in which the sick Russian was permitted to travel! How many caught small-pox from those cars?

The idea prevails among people in this section of the country, as doubtless in other places, also, that "*varioloid*" is not small-pox. Persons suffering from the disease in this mild form were found walking about and freely mixing with their neighbors during the "*scabby stages*," in total ignorance of the risk to themselves and to others! That word "*varioloid*" is a term not only bad but unfortunate, and should be dropped without delay.

Sioux City, March 1, 1881.

